# Synthetic artificial intelligence using generative adversarial network for retinal imaging in detection of age-related macular degeneration

**DOI:** 10.3389/fmed.2023.1184892

**Published:** 2023-06-22

**Authors:** Zhaoran Wang, Gilbert Lim, Wei Yan Ng, Tien-En Tan, Jane Lim, Sing Hui Lim, Valencia Foo, Joshua Lim, Laura Gutierrez Sinisterra, Feihui Zheng, Nan Liu, Gavin Siew Wei Tan, Ching-Yu Cheng, Gemmy Chui Ming Cheung, Tien Yin Wong, Daniel Shu Wei Ting

**Affiliations:** ^1^Duke-NUS Medical School, National University of Singapore, Singapore, Singapore; ^2^Singapore Eye Research Institute, Singapore, Singapore; ^3^Singapore National Eye Centre, Singapore, Singapore; ^4^School of Medicine, Tsinghua University, Beijing, China

**Keywords:** synthetic artificial intelligence, generative adversarial network (GANs), age-related macular degeneration, fundus image, deep learning, human-in-the-loop (HITL), realism assessment

## Abstract

**Introduction:**

Age-related macular degeneration (AMD) is one of the leading causes of vision impairment globally and early detection is crucial to prevent vision loss. However, the screening of AMD is resource dependent and demands experienced healthcare providers. Recently, deep learning (DL) systems have shown the potential for effective detection of various eye diseases from retinal fundus images, but the development of such robust systems requires a large amount of datasets, which could be limited by prevalence of the disease and privacy of patient. As in the case of AMD, the advanced phenotype is often scarce for conducting DL analysis, which may be tackled via generating synthetic images using Generative Adversarial Networks (GANs). This study aims to develop GAN-synthesized fundus photos with AMD lesions, and to assess the realness of these images with an objective scale.

**Methods:**

To build our GAN models, a total of 125,012 fundus photos were used from a real-world non-AMD phenotypical dataset. StyleGAN2 and human-in-the-loop (HITL) method were then applied to synthesize fundus images with AMD features. To objectively assess the quality of the synthesized images, we proposed a novel realness scale based on the frequency of the broken vessels observed in the fundus photos. Four residents conducted two rounds of gradings on 300 images to distinguish real from synthetic images, based on their subjective impression and the objective scale respectively.

**Results and discussion:**

The introduction of HITL training increased the percentage of synthetic images with AMD lesions, despite the limited number of AMD images in the initial training dataset. Qualitatively, the synthesized images have been proven to be robust in that our residents had limited ability to distinguish real from synthetic ones, as evidenced by an overall accuracy of 0.66 (95% CI: 0.61–0.66) and Cohen’s kappa of 0.320. For the non-referable AMD classes (no or early AMD), the accuracy was only 0.51. With the objective scale, the overall accuracy improved to 0.72. In conclusion, GAN models built with HITL training are capable of producing realistic-looking fundus images that could fool human experts, while our objective realness scale based on broken vessels can help identifying the synthetic fundus photos.

## Introduction

Age-related macular degeneration (AMD) is one of the leading causes of vision impairment in the elderly population globally. The Age-Related Eye Disease Study (AREDS) classified AMD into non, early, intermediate and advanced AMD (1). A meta-analysis of 129,664 individuals from 39 studies showed that the pooled prevalence of early, late and any AMD to be 8.01, 0.37, and 8.69%, respectively. By 2040, the number of people with AMD worldwide is projected to be 288 million (2). Early screening and detection of those at risk is crucial to prevent vision loss. However, the screening of AMD is limited by the availability of human assessors, coverage of screening programs and financial sustainability (3). With the aging population, there is an urgent clinical need to have an effective system to screen these patients for further evaluation.

In Ophthalmology over the last few years, deep learning (DL) systems with promising diagnostic performance have been developed to detect different eye diseases, such as diabetic retinopathy (DR) (4–8), glaucoma (9), AMD (10, 11) and retinopathy of prematurity (ROP) (12), showing substantial potential for improving healthcare ecosystems and implementation in screening programs (13, 14). The development of such robust DL systems requires a large amount of data for understanding specific scenarios and for developing effective applications, which is especially the case for the biomedical domains. However, collecting significant amounts of data might be challenging due to the substantial cost of performing screenings, as well as the low prevalence of certain diseases. The lack of large enough datasets can therefore hinder AI model development. More importantly, personal information of patients must be used under rigorously controlled conditions and in accordance with the best research practices (15). However, major problems remain in that medical records cannot be easily anonymized, and consent cannot be easily obtained for large populations (16–18). In addition, the availability of the more severe phenotypes of disease, such as intermediate and advanced AMD, may be too limited for training a DL system. In fact, while the current AI system (5) used for DR, glaucoma and AMD screening can detect eyes with DR very accurately, further enhancement of the AMD-suspect detection algorithm is required because the actual performance may not yet meet clinically acceptable metrics when tested on external validation datasets. For this reason, it is desirable for the models to be trained or fine-tuned with larger or additional datasets containing advanced AMD images.

Recent development in AI has offered an innovative alternative to the use of large datasets of patients’ images, by using real image datasets to artificially create synthetic images via DL frameworks, such as generative adversarial networks (GANs) (19). GANs are based on a game theoretic approach with the objective being to find Nash equilibrium between two networks, a generator (G) and a discriminator (D). The idea is to sample from a simple distribution, and then learn to transform this noise to the distribution of the data, using universal function approximators such as convolutional neural networks (CNNs), by adversarial training of G and D. The task of G is to generate natural looking images and the task of D is to decide whether the image is fake or real.

This study used a real-world non-AMD phenotypic dataset, which is from a population-based diabetic retinopathy screening cohort that has a limited number of advanced AMD images and applied GANs to artificially create more AMD positive images. Although GANs can be used to address the issue of limited access to large datasets, the development of GANs is itself data intensive. For example, if the training datasets contain only a small number of advanced AMD images, it is unlikely that the GAN model can produce an acceptable diversity of advanced AMD images. We therefore adopted a novel method called human-in-the-loop (HITL) to tackle this issue, which is defined as “algorithms that can interact with agents and can optimize their learning behavior through these interactions, where the agents can also be human” (20). We introduced human guidance during the training process and manually selected acceptable synthetic data generated by the GAN model, to feed back to the training loop. In addition, there is a lack of consensus on how to assess the outputs of GANs, particularly through qualitative assessment (21). To allow objective evaluation of the synthetic images, we proposed an objective realness scale based on how frequent the broken vessels are observed in the fundus images. The aim of this study is to use GAN to synthesize retinal fundus images with AMD features. We hypothesize that the synthetic fundus photos would not be easily discriminated from the real ones by human graders, and the use of an objective realness scale can improve the accuracy of discerning real versus synthetic images.

## Materials and methods

### Datasets

The GAN model was developed using 125,012 macula-centered fundus images from 67,867 patients from the Singapore Integrated Diabetic Retinopathy Program (SiDRP) 2016–2017 (Table 1). SiDRP (22) is a national DR screening program established in 2010, progressively covering all 21 primary care Polyclinics across Singapore, screening around a quarter of the population with diabetes annually. For each patient, two retinal photographs (optic disk- and macula-centered) are taken of each eye using Topcon TRC-NW8 Non-Mydriatic Retinal Cameras. SiDRP utilizes a tele-ophthalmology platform that transmits the digital retinal photography to a centralized team of trained professional graders for assessment of the fundus images. All the retinal images were graded using the AREDS classification of no, early, intermediate, and advanced AMD by experienced graders in the Ocular Reading Center of the Singapore National Eye Center. All advanced AMD images based on graders’ results were extracted and reviewed by two ophthalmologists. Any discordant gradings between the two were arbitrated by a senior ophthalmologist. We used 80% of the available data from SiDRP 2016–2017 for training and the remaining 20% for validation of the GAN model. Data from SiDRP 2018 was used for testing. This project did not involve patient interaction, therefore ethical approval was exempted by the SingHealth Institutional Review Board.

**TABLE 1:** Summary of training and validation dataset with Age-Related Eye Disease Study (AREDS) distribution.

SiDRP year	AREDS 0 (class 0) no AMD	AREDS 1 (class 1) early AMD	AREDS 2 (class 2) intermediate AMD	AREDS 3 (class 3) advanced AMD	Total number of fundus images	Number of patients
**Before macular segmentation**						
2016 and 2017	90,126	31,634	3,101	151	125,012	67,867
2018	41,757	14,023	1,319	95	57,194	33,455
**After macular segmentation**						
2016 and 2017	86,018	30,661	2,846	83	119,608	65,680
2018	39,612	13,452	1,191	48	54,303	29,857

AREDS 0 = no AMD, AREDS 1 = early AMD, AREDS 2 = intermediate AMD, AREDS 3 = advanced AMD. Images that do not have a round border after cropping were excluded.

### Pre-processing of fundus images

The retinal photographs had an original resolution of 3216 × 2136 pixels, and after the central retinal circle was extracted to a square template image, the template images were then rescaled to 1024 × 1024 pixels. The images were then normalized such that the disk is on the right side of the image, by horizontally flipping all images with the disk detected to be on the left side, as detected by an existing right/left eye DL model (23).

**FIGURE 1:**
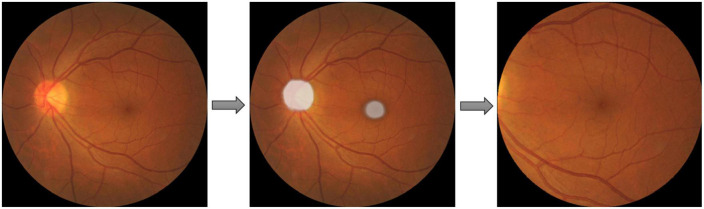
Macular segmentation example. Two U-net models were trained and combined to segment the optic disc (OD) and macula as ellipses shown in the second picture. The final step was to extract the circular two optic disc diameters (DD) macula region, as defined by the macula center, and the radius of 2DD.

AMD could be diagnosed by examining the region within two optic disk diameters of the macula (2DD Macula). Furthermore, the convincing synthesis of retinal vascular structure has proven to be challenging even with state-of-the-art GAN architectures from our preliminary work. Therefore, the extraction of this 2DD Macula region for GAN synthesis is desirable since this region tends to contain the requisite AMD features but leaves out much of the vascular structure complexity. Therefore, a U-Net model was applied to extract the macula region of the fundus images. Pixel-level annotated images were used to train U-Net DL models, that directly learn the optic disk localization and shape, and macula localization, end-to-end from retina images and their corresponding pixel-level annotations. A total of about 1,150 images from the SiDRP dataset of all AMD classes were annotated manually by an optometrist. An ellipse approximately covering the disk and a dot at the center of macula was annotated as the ground truth for each image. Two separate U-Net models were trained, one focusing on optic disk segmentation, and the other on macula segmentation. The outputs of these U-Nets could then be segmented and combined, to produce the optic disk and macula segmentations for new images. The final step was to extract the circular 2DD macula region, as defined by the macula center, and the radius of 2DD. An example of the segmentation process was illustrated in Figure 1. The macular region was then extracted from this template image to a 512 × 512 macular image, which was used in the GAN development and validation. Images that do not have a round border after macular segmentation, either resulted from inaccurate identification of the macular center or the original images being off centered, were excluded from the training dataset (Table 1).

### GAN iterative modeling and human-in-the-loop training

StyleGAN2 was used for the development of our AMDGAN model. StyleGAN2 is designed to be able to synthesize unique realistic images in some domain, given training examples of images in that domain. It further incorporates features such as the use of a mapping network to transform the latent vector before its usage as input to various levels of the generator, skip and residual connections (24, 25). Default hyperparameters (baseline learning rate = 0.002, minibatch size = 32, optimizer beta1 = 0.0, beta = 0.99, epsilon = 1e−8 etc.) were used.

**FIGURE 2:**
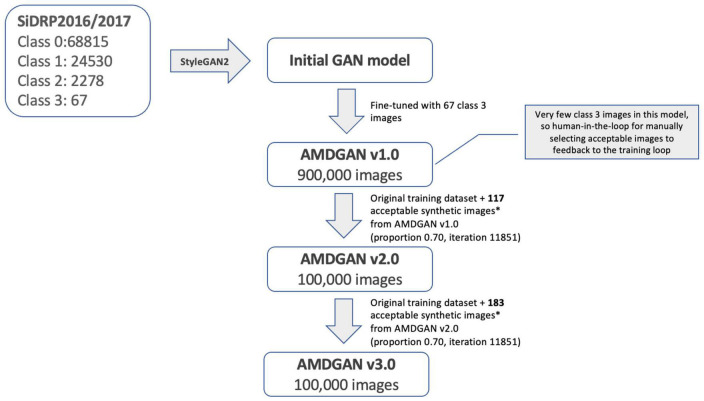
Development of AMDGAN models with human-in-the loop training. Acceptable synthetic images were defined as having realness score ≤1 (likely real and possibly real) and AREDS score = 3 (advanced AMD), which were manually selected from 5,000 synthetic images randomly drawn from AMDGAN v1.0 and v2.0.

The development of AMDGAN models is summarized in Figure 2. The initial GAN model was built using 95,690 fundus images of different AMD classes with macular segmentation from SiDRP 2016–2017, and minimum Frechet Inception Distance (FID) score was obtained after 11,731 iterations. Due to the relatively small percentage of advanced AMD images in a diabetic screening dataset, the 67 advanced AMD images were used to fine-tune the initial GAN model to generate AMDGAN v1.0. During the finetuning process, three iterations and three proportions of real to synthetic images were attempted, which gave nine combinations of different parameters with 100,000 images produced under each combination (details described in the Supplementary material).

**FIGURE 3:**
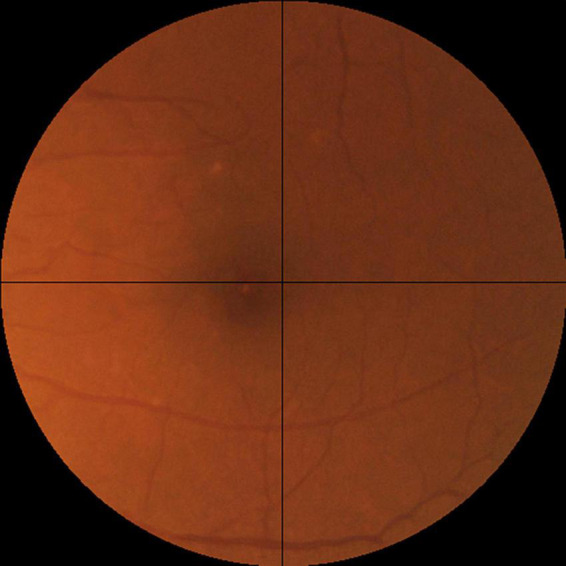
The objective realness scale. The macular segmented fundus image was divided into four quadrants, and the realness score was graded according to the following scale, Likely real (realness score = 0): broken vessels seen in ≤1 quadrant (25% of the image). Possibly real (realness score = 1): broken vessels seen in >1 but ≤2 quadrants (50% of the image). Likely synthetic (realness score = 2): broken vessels seen in >2 quadrants (75% of the image). Following this scale, the example image here has a realness score of 0 due to the broken vessels seen in the left upper quadrant.

For the assessment of realness, we observed that broken retinal vessels are the main feature that differentiates a synthetic image from a real one. We therefore proposed an objective scale based on the how frequently the broken vessels are observed in the four quadrants of a fundus photo (Figure 3). Likely real (realness score = 0) means broken vessels could be seen in ≤1 quadrant (25% of the image), possibly real (realness score = 1) means broken vessels seen in >1 but ≤2 quadrants (50% of the image), and likely synthetic (realness score = 2) means broken vessels seen in >2 quadrants (75% of the image).

Through manual grading of 5,000 randomly selected synthetic images from AMDGAN v1.0, 117 images that are AREDS grade 3 (advanced AMD) and have realness ≤ 1 (likely real and possibly real) were selected as acceptable images by an optometrist. The 117 images and the original training datasets were fed back into the training loop to build AMDGAN v2.0. The process was repeated to build AMDGAN v3.0 with the original training datasets and 183 acceptable images from AMDGANv2.0 (Figure 2). The FID score for AMDGAN v3.0 is 6.8084.

### Experiment 1: structural similarity index measure

To establish that the synthetic images are not just copies of training images, comparison of a subset of synthetic images to training images by the structural similarity index measure (SSIM) in a pairwise manner was conducted (26, 27). SSIM is a perceptual metric that measures the perceptual difference between two images based on luminance, contrast, and structure. The higher the SSIM, the more similar the pair of images are, with identical images having an SSIM of 1.00. For each of the four AMD classes (no, early, intermediate, and advanced AMD), five synthetic images were selected at random (with their AMD class determined by human grading). Then, for each of these synthetic images, its SSIM score was computed against each of the 95,690 training (real) images and compared with all three-color channels (red, green, and blue).

### Experiment 2: assess the diversity of AMD positive images in each GAN model

To assess if our AMDGAN models are capable of producing fundus images of different AREDS grades (i.e., the diversity), we trained an AMD classifier to automatically grade the images based on the AREDS classification, using real images from SiDRP 2016–2017, after the images were transformed and pre-processed in the same way as for training the AMDGAN models. Eighty percent of the data was randomly selected and used to train a VGG-19 classifier from ImageNet weight initialization, with 20% held out for internal validation. The classifier was trained to convergence over 200,000 iterations, with a batch size of 32, and a base learning rate of 0.001. The AMD classifier was then used to label 1,000 randomly selected synthetic images from AMDGAN v1.0, v2.0, and v3.0. The number of images under each AREDS grade was compared for three versions of AMDGAN model.

### Experiment 3: validation of the final GAN model via real versus synthetic grading

**FIGURE 4:**
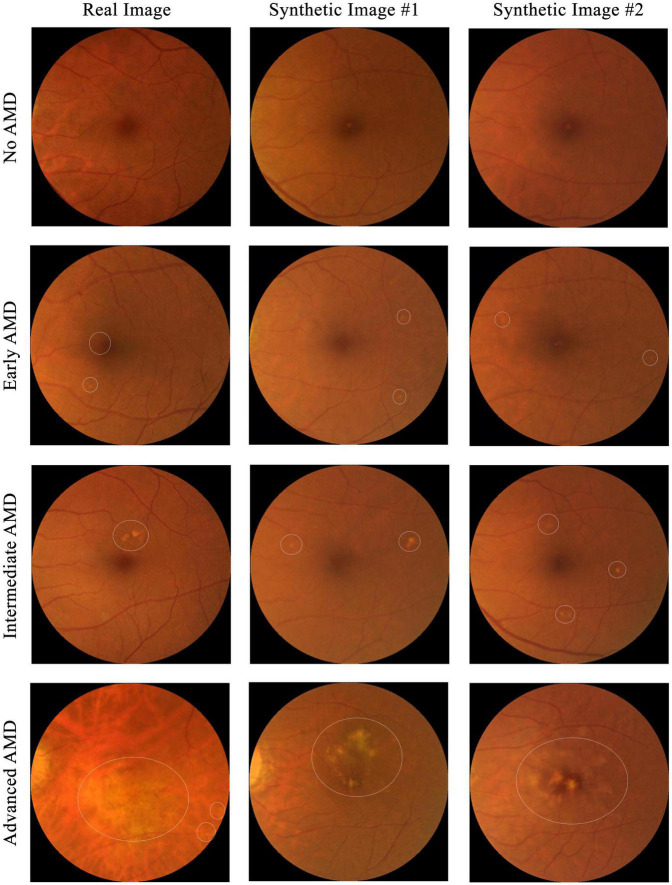
Examples of images used for the real versus synthetic grading. Images under the first column are real fundus images from SiDRP dataset and images under the second and third columns are synthetic images from our AMDGAN models. AMD areas on the fundus images are marked by the white circles.

To test if the synthetic images could be discriminated from the real ones, four ophthalmology residents were invited to manually annotate 300 images, which included equal numbers of real and synthetic images, with some examples shown in Figure 4. The 150 real images were randomly selected from SiDRP 2018 dataset, with 50 no AMD (class 0), 25 early AMD (class 1), 25 intermediate AMD (class 2) and 50 advanced AMD (class 3) images. The 150 synthetic images are composed of 50 no AMD images randomly selected from the initial AMDGAN model, 25 early AMD images from the AMDGAN model fine-tuned with training images of early AMD, 25 intermediate AMD images from the model fine-tuned with training images of intermediate AMD, and 50 advanced AMD images from the AMDGAN v3.0. For Classes 1 to 3 synthetic images, an initial manual filtering of the generated synthetic images was performed to ensure the images are of correct AREDS grade, and then the required number of images (25/25/50 for Classes 1/2/3) was randomly selected from the filtered set. Two rounds of grading were conducted. On the first round, ophthalmology residents were asked to label the images as likely real, possibly real, and likely synthetic based on their impression. After all residents completed the first round, they were given the objective realism scale based on broken vessels (Figure 3) to grade the same set of images but randomized to different orders. The residents were not aware of the number of real and fake images. All the gradings were done in dim environment based on original image size without zooming in. The software used to open images was Photos in MacBook and Windows Photo Viewer in Windows. Screen brightness was adjusted according to their preference.

### Statistical analysis

The statistical software used was R language (R V.3.5.3, R Foundation for statistical computing 2019, Vienna, Austria). The statistical analysis for the real versus synthetic grading experiment was done using metrics including accuracy, sensitivity, specificity, Area under the Curve of Receiver Operator Characteristic (AUC) and Cohen’s kappa score (κ score). When comparing to the binary ground truth (real or synthetic), likely real equals to real, possibly real and likely synthetic equal to synthetic. For the calculation of sensitivity, specificity and accuracy, true positive was defined as synthetic images being correctly graded as synthetic. The overall performance was analyzed via majority vote with the tied results arbitrated by an ophthalmologist. Cohen’s κ score was calculated by comparing each grader’s results to the ground truth.

## Results

### Experiment 1: structural similarity index measure

**FIGURE 5:**
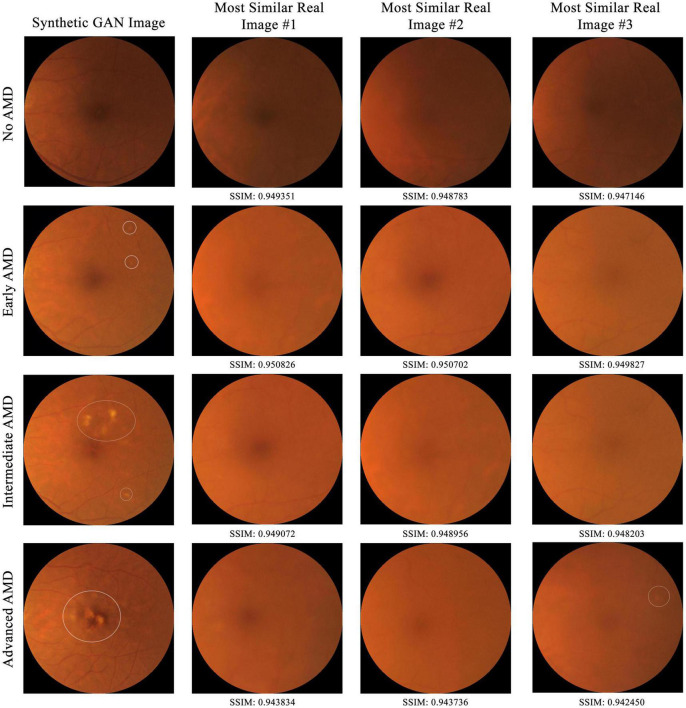
Comparison of a subset of synthetic GAN images to all real training images from SiDRP 2016–2017 dataset by the structural similarity index measure (SSIM) in a pairwise manner. The higher the SSIM, the more similar the pair of images are, with identical images having an SSIM of 1.00. Synthetic images with highest SSIM score under each AMD class are shown in this figure. AMD areas on the fundus images are marked by the white circles.

From the 20 randomly selected synthetic images, their highest SSIM scores when compared individually against all images from the training set are 0.949351, 0.950826, 0.949072, and 0.943834 for class 0 to 3 respectively. As shown in Figure 5, in no case do virtually identical real images exist in the training dataset. This suggests that the AMDGAN model indeed generates novel images, instead of simply memorizing and regurgitating existing images.

### Experiment 2: assess the diversity of AMD positive images in each GAN model

For the 1,000 synthetic images randomly drawn from the three versions of AMDGAN models, the number of images under each AREDS class labeled by the AMD classifier (AMDCLS) is summarized in Table 2. A balanced sensitivity/specificity of 0.76/0.76 was achieved for the classification of advanced AMD on a validation dataset from SiDRP 2018. The number of images with advanced AMD features increased from zero to 543 after two rounds of HITL training.

**TABLE 2:** Diversity of GAN-synthesized images by three versions of AMDGAN models.

	No AMD	Early AMD	Intermediate AMD	Advanced AMD
AMDGAN v1.0	19	293	688	0
AMDGAN v2.0	2	37	470	491
AMDGAN v3.0	9	65	383	543

The AREDS classes of the 1000 synthetic images from each version were labeled by an AMD classifier.

### Experiment 3: validation of the final GAN model via real versus synthetic grading

**FIGURE 6:**
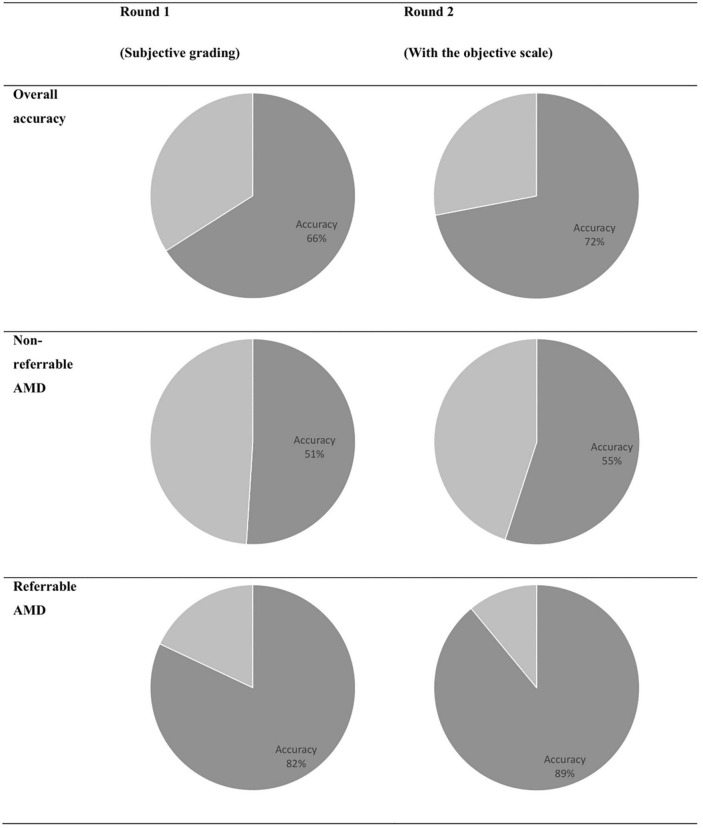
Overall accuracy of discriminating real and synthetic images.

The results of discriminating real from synthetic images are shown in Table 3. For the first round of grading, the sensitivity ranges from 0.33 to 0.76 and the specificity ranges from 0.41 to 0.94 among the four residents. When graded based on the objective scale, a substantial increase in specificity was observed with three residents, while the sensitivity remains about the same as round one. When comparing with the ground truth, slight to fair agreement was observed between the residents’ gradings and the ground truth, as evidenced by the κ score of 0.073–0.287. With the help of the objective scale, an increase in κ score was noted in three of the four residents, ranging from 0.200 to 0.700. The overall accuracy and the accuracy on discriminating different classes of real and synthetic AMD images were demonstrated by the pie charts in Figure 6. On the first round, the overall accuracy was 0.66, which increased to 0.72 with the objective realness scale. When breaking down to the non-referable AMD (no AMD and early AMD) classes, the accuracy in the first round was close to chance (0.51), which increased to 0.55 with the objective scale on the second round. For the referable AMD classes (intermediate AMD and advanced AMD), residents could discriminate synthetic images from the real ones with an accuracy of 0.82 and 0.89 for the first and second round of grading, respectively.

**TABLE 3:** Results of the real versus synthetic grading by four ophthalmology residents.

	Round one (subjective grading)					Round two (Objective grading)				
	**Sensitivity (95% CI)**	**Specificity** **(95% CI)**	**Accuracy** **(95% CI)**	**AUC** **(95% CI)**	**κ**	**Sensitivity** **(95% CI)**	**Specificity** **(95% CI)**	**Accuracy** **(95% CI)**	**AUC** **(95% CI)**	**κ**
Resident 1	0.48 (0.40, 0.56)	0.81 (0.73, 0.87)	0.64 (0.59, 0.70)	0.64 (0.59, 0.64)	0.287	0.49 (0.40, 0.57)	0.99 (0.96, 1.00)	0.74 (0.69, 0.79)	0.74 (0.70, 0.74)	0.480
Resident 2	0.67 (0.59, 0.74)	0.41 (0.33, 0.49)	0.54 (0.48, 0.59)	0.54 (0.49, 0.54)	0.073	0.57 (0.49, 0.65)	0.71 (0.63, 0.78)	0.64 (0.59, 0.70)	0.64 (0.59, 0.64)	0.287
Resident 3	0.33 (0.25, 0.41)	0.94 (0.89, 0.97)	0.63 (0.58, 0.69)	0.63 (0.59, 0.63)	0.267	0.43 (0.35, 0.52)	0.77 (0.69, 0.83)	0.60 (0.54, 0.66)	0.60 (0.55, 0.60)	0.200
Resident 4	0.76 (0.68, 0.83)	0.43 (0.35, 0.51)	0.59 (0.54, 0.65)	0.59 (0.54, 0.59)	0.187	0.79 (0.71, 0.85)	0.91 (0.86, 0.95)	0.85 (0.80, 0.89)	0.85 (0.81, 0.85)	0.700
Non-referrable AMD	0.21 (0.13, 0.32)	0.81 (0.71, 0.89)	0.51 (0.43, 0.60)	0.51 (0.45, 0.51)	0.030	0.13 (0.07, 0.23)	0.97 (0.91, 1.00)	0.55 (0.47, 0.63)	0.55 (0.51, 0.55)	0.110
Referrable AMD	0.80 (0.69, 0.88)	0.84 (0.74, 0.91)	0.82 (0.75, 0.88)	0.82 (0.75, 0.82)	0.640	0.79 (0.68, 0.87)	0.99 (0.93, 1.00)	0.89 (0.82, 0.93)	0.89 (0.84, 0.89)	0.770
Overall performance	0.52 (0.44, 0.60)	0.80 (0.73, 0.86)	0.66 (0.60, 0.71)	0.66 (0.61, 0.66)	0.320	0.45 (0.37, 0.53)	0.99 (0.95, 1.00)	0.72 (0.66, 0.77)	0.72 (0.68, 0.72)	0.430

Round one was done based on graders’ subjective impression and round two was done based on the objective scale.

## Discussion

This study used a large real-world dataset of 125,012 fundus photos to test if GAN could produce synthetic fundus images with AMD lesions that look realistic, when real AMD images are limited in the training dataset. Due to the naturally low percentage of advanced AMD images in a real-world dataset, our initial GAN model (AMDGAN v1.0) did not produce satisfactory examples with AMD features, particularly the advanced AMD ones. To overcome this limitation, we introduced human guidance to the training process (HITL method) via manually selecting images with balanced realness and AMD features to train a secondary model, which has not been reported in the field of fundus image synthesis using GANs. Through HITL training, the percentage of AMD positive images increased after one and two rounds of HITL training. In addition, the SSIM scores gave quantitative assessment to support the observation that our GAN models could produce novel images that are not just replicas of the real images. Despite high SSIM score of up to 0.9508, the synthetic image does not resemble the most similar real image in the dataset. Besides, the FID score of AMDGAN v3.0 model is 6.8084. FID is a metric used to assess the quality of images created by a generative model, by comparing the distribution of a sample of generated images, with the distribution of a set of real images. The smaller the FID score value, the closer the two distributions, and thus the more realistic the generated synthetic images are to actual real images in general (28). The value of FID and SSIM experiment demonstrated that our AMDGAN models are capable of generating synthetic images that are similar to real ones, yet not reproducing them.

Generative Adversarial Networks have been applied in both medical and non-medical fields, such as image synthesis, image to image translation, text to image translation, super resolution, segmentation, classification, and music composition (29–31). One of the main applications of GANs in the medical field is image synthesis, including various image modalities such as breast ultrasound (32), mammograms (33), computed tomography (CT) (34–37), magnetic resonance images (MRI) (38), cancer and pathology images (39). In ophthalmology, several adversarial learning models for generating fundus images and Optical Coherence Tomography (OCT) images with and without pathology have been reported, including (1) generating synthetic retinal blood vessel trees and translating back to a raw image (40–42); (2) combination of vessel tree, optic disk images to generate normal color fundus photos (43); (3) synthesizing fundus images of AMD (44), glaucoma (45), DR (46) and ROP (47); (4) using GAN-synthesized OCT images to train a DL framework for detecting cases that require urgent referral (48); (5) predicting the post-treatment OCT images of patients receiving anti-vascular endothelial growth factor (anti-VEGF) (49, 50); (6) cross-modality image synthesis using fundus photographs to produce fluorescein angiography (51). However, the clinical use cases of GANs, such as training and validation of DL systems, are yet to be firmly established (21, 52).

Before introducing synthetic data to the development of DL systems, evaluating the outputs of GANs using qualitative and quantitative measures are critical. Quantitative methods generally do not involve human assessment. Examples include the inception score (IS) to classify the synthetic samples with a discriminative model trained on real ImageNet dataset, and SSIM to compare if the synthetic images are merely replicates of the real images (53, 54). On the other hand, qualitative assessment generally relies on subjective human judgment for the realness and gradability of the synthetic outputs, especially for biomedical images. To allow more consistent qualitative measure and perhaps comparison between different GAN models, we proposed a novel objective realness scale based on the frequency of broken vessels in the retinal fundus images. In our experiment, the introduction of the objective realness scale helped to improve the residents’ performance to discriminate real from synthetic images, as evidenced by the increase in the overall accuracy, specificity, and kappa score. However, when grading is based on broken retinal vessel alone, some synthetic images might be graded as real even though they have other synthetic features, such as pixelated retina pigmented epithelium at the fovea and abnormally straight temporal vascular arcade. This observation indicates that broken vessels are a specific feature of synthetic images but using this feature alone may lead to misclassification of some synthetic images as real ones, resulting in higher false negative errors and thus the decrease in sensitivity.

In terms of realism assessment, the image grading performed by ophthalmology residents demonstrated that the GAN-generated synthetic fundus photos could imitate the real ones with AMD lesions. When the ophthalmology residents were asked to discern whether an image is real or synthetic based on their impression, the accuracy ranges from 0.54 to 0.64. Similar results were reported by Burlina et al. (44) using 133,821 AMD fundus images from AREDS to build two ProGAN models to synthesize non-referable and referable AMD images, respectively. Two retinal specialists had accuracies of 59.5 and 53.7% at discriminating real from synthetic images (44). In addition, using 4,282 pairs of fundus images and retinal vessel maps from a ROP screening program, Chen et al. (52) built a GAN model by tunning pix2pixHD with segmented vessel map and fundus images. The synthetic images generated by their model could fool four ophthalmologists at accuracies of 49–61% (52). In our study, when the real vs. synthetic results were stratified to referable and non-referable AMD images, we observed that real and synthetic non-referable AMD images appear equally real to the residents (accuracy of 0.51), while synthetic referable AMD images were much more easily identified (accuracy of 0.82). This observation likely arises from the imbalanced distribution of images under each AMD class in the training datasets, which had 93,345 non-referable AMD images but only 2,345 referable AMD images. The results again addressed the data-intensive nature of GANs, which requires sufficient training data exhibiting the desired underlying class and representative variability.

Despite the increase in the proportion of synthetic images with AMD lesions, we observed that some synthetic images of advanced AMD share similar pathological patterns. The limited number of advanced AMD images in the training datasets and the acceptable images added to the training loop is likely the reason for repetitive features observed in the outputs of our GAN model. Although GAN models have been successfully built to synthesize realistic faces, even with a small training dataset of around 100 faces, retinal fundus images seem to be more challenging to synthesize, in particular abnormal examples with disease conditions (55, 56). The difficulty may arise from the fact that the retinal vasculatures and pathological lesions do not have roughly fixed landmarks like the faces, in which the location of eyes, nose, and mouth could augment the development of respective GAN models (57, 58). Despite the fact that GAN was proposed to augment small training datasets by artificially producing more synthetic images (19, 59, 60), the development of GANs for synthesizing retinal images with pathological features is still data-intensive, and the least amount of training data required to build an effective GAN model remains unknown.

Although GAN may not be able to rectify a small training dataset of retinal images with pathological features due to low disease prevalence, it could still be a powerful tool for privacy preservation before data sharing. As demonstrated in our experiment, high quality fundus images of the non-referable AMD classes were synthesized by the GAN models, when the training datasets contain sufficient real images. A recent study from DuMont Schütte et al. (61) proposed an open benchmark to assess the quality of synthetic chest radiographs and brain CT scans from two GAN models, which indicated that the barriers to data sharing may be overcome by synthetic data. Future research could be attempted to build GAN models using datasets comprising mainly of AMD images and the GAN-synthesized images could be shared among different research groups as a training or independent external validation dataset, while preserving the privacy of the real dataset.

### Limitations

There are several possible limitations to the presented study. Firstly, the optimal distribution of real and synthetic images to be used to train the various AMDGAN iterations as to produce outputs with the most desirable diversity-realism tradeoff is unknown *a priori*. As such, several plausible proportions were attempted in the HITL models and the best amongst them selected, but it is not guaranteed that this is the ideal way to optimize the input training distribution. Second, the impact of adding the manually selected acceptable images to the training loop, such as the weightage, remains unknown. Third, the real versus synthetic grading experiment was conducted by four ophthalmologist residents. Inviting more senior ophthalmologists of various levels of experience may be more accurate on judging the realness of the fundus images. Another limitation is that more advanced versions of StyleGAN, such as StyleGAN3, have been released since this study was first commenced. However, since our human-in-the-loop methodology involves fine-tuning a StyleGAN model in response to human grader assessment, it is infeasible to incorporate new versions of StyleGAN without redoing the bulk of the study. Nonetheless the quality of images from StyleGAN2 was sufficient to demonstrate the potential of our method. Lastly, to use GAN produced synthetic images for the development and validation of DL systems, the ground truth of the synthetic images’ classes needs to be determined. For most of GAN related studies reported in the literature, the classes of the synthetic images are either labeled automatically by the GAN model or by a separate classifier. Whether the machine generated ground truth is reliable remains unknown and difficult to validate, because it is challenging and time-consuming to produce human-validated ground truth due to the large number of images.

### Potential clinical impact

Despite the challenges of building a powerful GAN model as discussed above, we still see several potential areas of application within the clinical space. First of all, GANs are capable of producing realistic medical images without replicating the real training images. As a result, GAN-synthesized images with enhanced diversity could be used for medical education purposes. In addition, future work could be attempted to identify the minimal number of images with pathological features required to train an effective model, which is likely to be useful for developing DL frameworks for detecting rare diseases. Last but not least, GANs synthesized data could be used within a “sandbox,” which enables a computer security mechanism and allows opening files, testing models or programs in an isolated environment without affecting the system on which it runs. The sandbox environment was described by the UK’s Financial Conduct Authority in 2015 for regulatory purposes as “a “safe space” in which businesses can test innovative products, services, business models and delivery mechanisms without immediately incurring all the normal regulatory consequences of engaging in the activity in question,” which was used to constructively engage innovators, and to remove unnecessary barriers to innovation (62). In healthcare, sandbox has been adapted for outcome-focused purposes, such as testing how diagnostic DL systems affect patient outcomes, and for data-focused purposes, such as facilitating access to health data for development and testing of new technologies (63). Therefore, synthetic data from GAN models is likely to be beneficial for the application of sandbox in healthcare.

## Conclusion

Our GAN models trained using a non-AMD phenotypical dataset can generate synthetic images that are not easily discerned from the real ones to human eyes, in particular for non-referable synthetic AMD images. However, the development of GAN models remains data intensive and GANs may not be the best solution to rectify small training datasets for synthesizing realistic looking fundus images with intermediate and severe AMD lesions. Nevertheless, GAN could potentially be a powerful tool for data privacy preservation, which would allow data sharing across different research groups in the sandbox environment for the development or testing of the commercially available DL systems.

## Data availability statement

The datasets presented in this article are not readily available because the SiDRP dataset is available from the respective research institute, but restrictions apply to the availability of these data, which were used under agreement for the current study, and so we cannot make them publicly available. However, datasets and relevant data dictionaries will be made available upon reasonable request and with permission of the respective research institutes. Source code for the deep learning algorithms used in this study will also be made available from the authors upon request. Requests to access the datasets should be directed to DT, daniel.ting.s.w@singhealth.com.sg.

## Ethics statement

Ethical review and approval was not required for the study on human participants in accordance with the local legislation and institutional requirements. Written informed consent for participation was not required for this study in accordance with the national legislation and the institutional requirements.

## Author contributions

GT, C-YC, GC, TW, and DT contributed to the conception of this study. ZW, GL, and DT contributed to the study design, analysis, and interpretation of the data, responsible for the decision to submit the manuscript and had access and verified the underlying study data. ZW, GL, WN, T-ET, JaL, SL, VF, JoL, and LS contributed to the data acquisition. All authors contributed to drafting and revising the manuscript, had access to all the data, and contributed to the article, and approved the submitted version.
