# Supplementary material for: Synthetic artificial intelligence using generative adversarial network for retinal imaging in detection of age-related macular degeneration
Source: Front Med (Lausanne). 2023 Jun 22;10:1184892. doi: 10.3389/fmed.2023.1184892 (PMC10324667; doi:10.3389/fmed.2023.1184892)
Supplement: Supplementary file 1 [file Data_Sheet_1.docx]

**Supplementary material**

**GAN Iterative Modeling**

The initial GAN model was built using 95,690 fundus images of different AMD classes with macular segmentation from SiDRP 2016-2017, and minimum Frechet Inception Distance (FID) score was obtained after 11731 iterations. Due to the relatively small percentage of advanced AMD images in a diabetic screening dataset, the 67 advanced images were used to fine-tune the initial GAN model to generate AMDGAN v1.0. The first three iterations were chosen, namely 11791, 11851 and 11911 iterations, with 60 iterations increment, because further increase of iterations leads to more focus on AMD lesions at the cost of more broken vessels. In addition, synthetic images were generated with three proportions of 0.50, 0.70 and 0.90. For example, proportion 0.70 means that when fine-tuning the images, there is 0.70 chance of picking up an image from the advanced AMD pool (67 images), 0.30 chance of picking up an image from the non-advanced AMD pool (80%/10%/10% from no AMD, early AMD, and intermediate AMD respectively). With three iterations and three proportions, nine combinations were used to generate synthetic images. For AMDGANv1.0, in total 900,000 images were synthesized with 100,000 images under each combination. By comparing the first 1,000 images of each combination, we concluded that iteration 11851 with 0.70 proportion delivered the best balance between diversity of AMD lesions and realness of the images. The same parameters were used for AMDGAN v2.0 and v3.0.
